# Modulation of the honey bee queen microbiota: Effects of early social contact

**DOI:** 10.1371/journal.pone.0200527

**Published:** 2018-07-12

**Authors:** J. Elijah Powell, Daren Eiri, Nancy A. Moran, Juliana Rangel

**Affiliations:** 1 Department of Integrative Biology, University of Texas, Austin, TX, United States of America; 2 Department of Entomology, Texas A&M University, College Station, Texas, United States of America; Universiteit Leiden, NETHERLANDS

## Abstract

As the sole reproductive female in a honey bee (*Apis mellifera*) colony, the queen’s health is critical to colony productivity and longevity. Beekeeping operations typically rely on the commercial mass production of queens for colony multiplication, which involves manipulating and isolating the queens by confining them in cages during early development. Using common queen-rearing techniques, this study shows that segregating newly eclosed queens from their worker attendants for 72 hours using queen protector cages has a significant impact on the total amount of gut bacteria carried by those queens compared to queens that have unrestricted access to attendants upon eclosion. Isolated virgin queens sampled immediately after isolation at 4 days post eclosure had significantly more bacteria and a less consistent microbiota composition than their non-isolated peers. Furthermore, this effect lasted into the mating life of queens, since mated queens that had been isolated after emergence and then sampled at 14 days post eclosure also had significantly more microbiota compared to non-isolated mated queens of the same age. The causes and potential impacts of this alteration are not clear and deserve further investigation. This study also verifies earlier findings that honey bee queens lack the core microbiome found within honey bee workers.

## Introduction

As highly eusocial insects, honey bees (*Apis mellifera*) live in colonies composed of one female queen who performs all reproductive tasks, tens of thousands of female workers, and a limited number of seasonal males, called drones [1,2]The queen passively regulates this extreme form of reproductive monopoly by releasing glandular pheromones [3–5], which are highly attractive to workers [6–10], inhibit queen rearing [11–13], and suppress worker ovary activation [14,15]. Because the queen is responsible for the production of workers, colony productivity is directly linked to the queen’s overall reproductive health [16,17].

Queen failure, which can occur due to pathogens [18,19], pesticide exposure [9,20], inadequate mating [6–8], or a combination of factors, has recently been reported as one of the top causes of colony losses in the U.S. [21–23]. To avoid sudden queen failure, many modern beekeepers have stopped relying on a colony’s natural queen replacement processes [2,24]. Instead, they prefer to rely on a systematic annual queen replacement procedure that employs queens raised en masse by commercial queen-rearing operations. Briefly, 1-day-old worker larvae are grafted into plastic “queen” cups and placed in queenless “cell-building” colonies where nurse workers feed them royal jelly until their cells are capped, after which they undergo pupation [2]. A few days before the queens are expected to eclose, beekeepers either move the cells individually into small “nucleus” colonies that contain a few hundred workers where young queens emerge and mate naturally, or they enclose the cells in protector cages and put them in queen “banking” colonies where young queens, either virgin or mated, are held for days or weeks until they are employed [25]. This banking process prevents queens from coming in direct contact with other queens, thus avoiding potential queen-queen elimination duels [25].

A similar isolation experiment performed with newly emerged honey bee workers showed that caging workers, which only allowed them to have contact with nestmates through trophallaxis, prevented normal colonization by the core gut microbiota [26]. This perturbation may consequently affect social immunity responses at the colony-level [27]. In workers, the core gut microbiome consists of eight core bacterial lineages that are transmitted through social interactions [26]. These lineages are highly consistent across age and geography [28] and contribute to digestion and development [29], potentially protecting the bees against pathogens [30]. Previous studies [31,32] have established that the microbiota of queens differs substantially from that of workers. Compared to workers, queens contain higher representation of bacterial lineages that are found in nectar, larvae, and the hive. These include certain Acetobacteraceae lineages (referred to as “Alpha 2.1” and “Alpha 2.2”) that are the same as, or closely related to, *Parasaccharibacter apium* [33]. How the gut microbiota of queens is affected by isolation within queen protector cages is currently unknown. This study is aimed at addressing the possible consequences of queen isolation on the composition of the gut microbiota, which may in turn affect overall queen and colony health.

We used quantitative PCR along with deep sequencing of amplicons of the V4 region of the bacterial 16S rRNA gene to characterize both the size and the composition of the microbiome of queens. We compared microbiome features at two points in queen development: virgin queens at 4 days post eclosure and mated queens at 14 days post eclosure. We compared queens that either maintained contact with their nurse bees throughout development (non-isolated, control group) or that were separated from their nurses for 72 hours at 24 hours post eclosure in a queen protector cage (isolated, experimental group). Since the latter treatment matches the caging method typically used for commercial queen production, our study provides new information on whether current queen-rearing methods may interfere with the development of the normal gut microbiota in honey bee queens.

## Materials and methods

### Experimental set up and queen rearing

Two honey bee colonies located at the Janice and John G. Thomas Honey Bee Facility of Texas A&M University in College Station, Texas were used for this study. Twenty 1-day-old worker larvae from each colony were grafted into individual queen cups and then transferred into a 5-frame cell-building colony, following standard queen-rearing procedures [25]. Once the queen cells were capped, they were placed individually in plastic protector cages. All queens that emerged inside the cages were placed individually into queenless, 2-frame nucleus colonies containing a few hundred workers and a small patch of brood of varying ages, as well as honey and pollen. Five additional queens were produced via the grafting method and were reared to adulthood without being caged. Once the latter five queens emerged from their cells they were stored in 1.5 mL microcentrifuge tubes with 95% ethanol at -20°C.

Because sampling bee guts relies on harvesting the entire gut through dissection, it is impossible to sample the same individuals repeatedly over time. Due to this constraint, we opted for sampling queens at two collection times: At 4 days of age, when the queens were still virgins, and at 14 days of age, after they had mated and had been observed laying worker-destined eggs. Adult queens placed in the nucleus colonies were separated into 4 treatment groups: (1) Isolated virgin queens (IVQs), (2) isolated mated (IMQs) queens, (3) non-isolated virgin queens (NVQs), and (4) non-isolated mated queens (NMQs). Isolated queens (IVQs and IMQs) remained inside the queen cages for four days post emergence. Non-isolated queens (NVQs and NMQs) were released from their cages 24 hours post emergence to increase their acceptance rate by workers in the nucleus colonies. Therefore, isolated and non-isolated queens differed by 3 days in their exposure to full body contact with nurses and hive materials. Virgin queens and mated queens were collected 4 and 14 days post emergence, respectively. All 14-day-old queens successfully mated (IMQs and NMQs), as verified by confirming the presence of worker-destined eggs in the brood area of the mating nucleus colonies. Our sampling did not separate the effects of mating and age. In practice, queens are mated by 14 days following eclosure, and we aimed to represent the normal stages of a queen’s life cycle.

**Fig 1:**
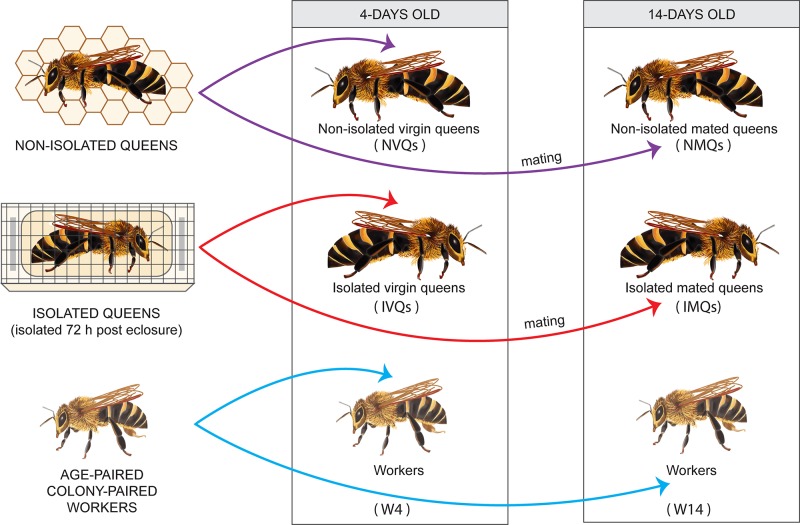
Experimental design. Schematic diagram of the experiments conducted to determine the effect of honey bee queen isolation post eclosure on the subsequent gut microbiota, and to compare queen and worker gut microbiota. Experimental details are given in the *Methods* section.

Additionally, 5 frames of capped brood (with no adult bees on them) from both source colonies were placed in an incubator overnight at 34°C. Newly emerged workers were labeled on the thorax with latex paint of different colors (Testors, Vernon Hills, Illinois, USA) to indicate each worker’s date of emergence and colony of origin. All workers emerged within twelve hours of all experimental queens. Approximately 20 newly emerged workers were placed inside each nucleus colony at the same time that a queen was introduced. Because the workers came from the same colony used for grafting, the workers were sisters to the queens. Marked workers were then collected simultaneously with the collection of queens, at 4 or 14 days old. For colony 2, we were unable to recover marked workers at 14 days, so these are only represented by a single colony. All queens and workers were placed in 1.5 mL microcentrifuge tubes or 15 mL conical tubes, respectively, and stored in 95% ethanol at -20°C until DNA was extracted. This experimental strategy is summarized in Fig 1.

### DNA extraction

DNA from the entire gut (from crop to rectum) of 45 queens and 20 workers was extracted under sterile conditions as described by Powell et al. [26] using a CTAB (cetyltrimethylammonium bromide) bead-beating method. Briefly, fresh bee guts from live bees were placed in ethanol. For processing, they were removed from the vials and placed individually in separate tubes to air dry. Five to 10 minutes later, 728 μL of CTAB and 20 of μL proteinase K (Sigma, St. Louis, MO, USA) was added to each tube, and the sample was homogenized with a pestle. The homogenate was pipetted into a fresh bead-beating tube with ~0.5 mL of 0.1-mm silica zirconia beads (Sigma, St. Louis, MO, USA). Then, 2 μL of 2-mercaptoethanol (Sigma, St. Louis, MO, USA) was added to each tube, which was bead beaten (BioSpec Products, Bartlesville, Oklahoma, USA) for 2 min at full speed, followed by 1 min on ice and a final 2 min of bead beating at full speed. The tubes were incubated at 56°C for 45 min before adding 2 μL RNase A (Invitrogen, Carlsbad, CA, USA), and were then incubated overnight. Approximately 600 μL of the digested lysate was added to a new tube with 600 μL of 25:24:1 phenol-chloroform-isoamyl alcohol (Ambion, Austin, TX, USA). The tubes were then inverted 5 times and placed on ice for 2 min before centrifuging at full speed for 15 min at 4°C. The aqueous phase was alcohol precipitated, washed, air-dried, and then resuspended in 50 μL of nuclease-free water. The extracted DNA was quantified using a Qubit Fluorometer (Invitrogen, Carlsbad, CA, USA) and normalized to 10 ng/μL with nuclease-free water.

### Diagnostic PCR

The presence of bacterial DNA in newly emerged queens and workers was verified using the universal bacteria 16S rRNA gene primers 27f-short and 1507r. To verify DNA quality, the samples were also screened with eukaryotic ef1α primers [34]. PCR screens were run with a negative (ddH_2_0) and a positive (adult *A*. *mellifera* gut DNA) control on a 2% agarose gel (120 V, 30 min). Samples that did not produce amplicons with the universal bacterial primers were not quantified using qPCR and were not used for Illumina sequencing.

### Bacterial abundance assessed by 16S rRNA gene copy number

Quantitative PCR was performed to assess 16S rRNA gene copy number within individual samples. We amplified the 16S rRNA gene using universal bacterial primers as described in Raymann et al. [35]. Statistical differences in the absolute abundance of 16S rRNA gene copy number were analyzed in R [36] by first performing a Shapiro-Wilk normality test. Once determined to have a normal distribution, comparisons between groups of queens and workers were performed with a one-way analysis of variance (ANOVA) test. Subsequent post-hoc pairwise analysis was done using the Tukey Honest Significant Difference (HSD) method multiple comparisons test.

### Illumina sequencing

PCR was performed to amplify the V4 region of the 16S rRNA in triplicate by using the 515F and 806R primers [37]. The reactions were then purified with AMPure XP Beads (Beckman Coulter, Indianapolis, Indiana, USA). The resulting cleaned amplicon was sent to the Genome Sequencing and Analysis Facility at the University of Texas at Austin for Illumina sequencing on the MiSeq platform (2x250 b.p. run). Raw read and read-processing statistics are reported in S1 Table. Individual samples that did not amplify, or those with low amounts of quality reads, were excluded from the dataset. Sample-specific information is reported in S2 Table and a summary of the distribution of sample types is included in S3 Table.

Because all the primers typically used to explore microbial diversity manifest varying levels of bias, the choice of primers affects proportions of lineages in the resulting amplicon library [38,39]. Recent studies have explored novel approaches like poly-A tailing with reverse transcrition of rRNA [40] or use of alternate targets like the *rpoB* gene [41]. The choice of the 16S rRNA gene primers in this study was largely guided by accumulated data concerning the bee gut microbiome based on 16S rRNA gene characterization; many of these previous studies utilized the same hypervariable region and primer sequences [28,34,42–48]. This primer choice allowed us to generate data that were easily comparable across studies.

### Nucleotide sequence accession number

All of the sequence files are available from the Sequence Read Archive (http://www.ncbi.nlm.nih.gov/sra), BioProject identifier (ID) PRJNA429464.

### Sequence analysis

Illumina sequence reads were processed with QIIME 1.9 [49]. Forward and reverse Illumina reads were joined using the SeqPrep method [50] via the join_paired_ends.py script with modified settings: the minimum allowed fraction of matching bases was set at 0.2, and maximum mismatched high quality bases were allowed to join reads at 0.06. Chimeric sequences were removed using the usearch6.1 detection method [51] implemented in the identify_chimeric_seqs.py script in QIIME. Operational Taxonomic Units (OTUs) were clustered at 97% and representative sequences were assigned via uclust [51]. Mitochondrial, chloroplast, singleton and reads containing multiple ambiguous basecalls were filtered from the dataset.

Taxonomy was assigned to OTU representative sequences using the SILVA SINA aligner (v1.2.11) [52]. Lactobacilli and Acetobacteraceae reads were further examined in order to properly assign taxonomy and to discriminate between host-associated and environmental lineages. Taxonomic placement is more difficult for those lineages than for the easily resolved host-associated groups like *Snodgrassella*, *Gilliamella*, and *Frischella* [53]. For OTUs of Lactobacilli and Acetobacteraceae, representative sequences were placed in alignments and were used to reconstruct maximum likelihood trees using the alignments and methods used by Cariveau et al. [53]. In brief, full length or near full length 16S rRNA sequences of bacteria associated with corbiculate bees (*Bombus* and *Apis*) and other closely related bacterial taxa were retrieved from Genbank and used along with the representative sequences from our dataset. For those binned as Acetobacteraceae by SILVA, the sequences were aligned via the Infernal aligner in RDP 10 [54], and the alignment was used to reconstruct maximum likelihood trees with bootstrap support via RAxML v7.4.2 [55]. A GTRgamma base substitution model was used with 1000 iterations. The *Lactobacillus* tree was inferred by a different route due to the complexity and size of the genus and ambiguities within 16S rRNA gene alignments [56]. A highly curated secondary structure model based alignment was used from a study on host specificity between hymenopterans and lactobacilli [57]. Lactobacilli representative sequences from our dataset were added to the alignment using PyNAST [58]. Resulting trees were visualized with FigTree 1.4.3 (http://tree.bio.ed.ac.uk/software/figtree/). Reads were then binned based on whether or not they had phylogenomic placements with host-associated lineages.

### Taxonomic analysis

The 44 most abundant OTUs, which comprised 96% of the filtered reads, were used for taxonomic analysis. The taxonomic assignments for these high abundance OTUs are included in S5 Table and the representative sequences are in S1 Appendix. These were combined into species and used to generate relative abundance plots. Absolute abundance of bacterial species within samples was obtained using the techniques described in Raymann et al. [35]. Briefly, the total 16S rRNA copies reported for an individual sample were multiplied by the relative abundance of a given taxon within that sample. This product was then divided by the number of 16S rRNA operons reported for that taxon based on complete, fully assembled genome sequences. Although strains may vary in 16S rRNA operon number, we expect this variation to be minor. The average relative proportions and absolute abundances of specific taxonomic lineages were compared statistically with Kruskal-Wallis one-way ANOVA tests from the R package pgirmess [59] and box plots were made in R using ggplot2 [60].

### Alpha and beta diversity

Alpha diversity was examined by calculating the Shannon’s Index of diversity and converting it to the linear Effective Number of Species [61]. This calculation was based on a subsampling method performed with 10 iterations at every 100 reads. A uniform depth of 1800 reads per sample was used, as this depth preserved a high number of samples for examination and showed saturation of alpha diversity (S2 Fig). To test alpha diversity between groups we first used the Bartlett test of homogeneity of variances, followed by a Welch’s ANOVA [36] and post hoc Games-Howell test for multiple comparisons [62]. For Beta diversity we used an OTU table subsampled at 1800 reads without replacement as a basis for analyzing Bray-Curtis distances. Principal Components Analysis (PCA) plots of Bray-Curtis distances were generated, and statistical differences between groups of queens and workers were examined with the R VEGAN Adonis method for permutational multivariate analysis of variance (PERMANOVA) tests [63,64].

## Results

### Total bacterial abundance and alpha diversity

**Fig 2:**
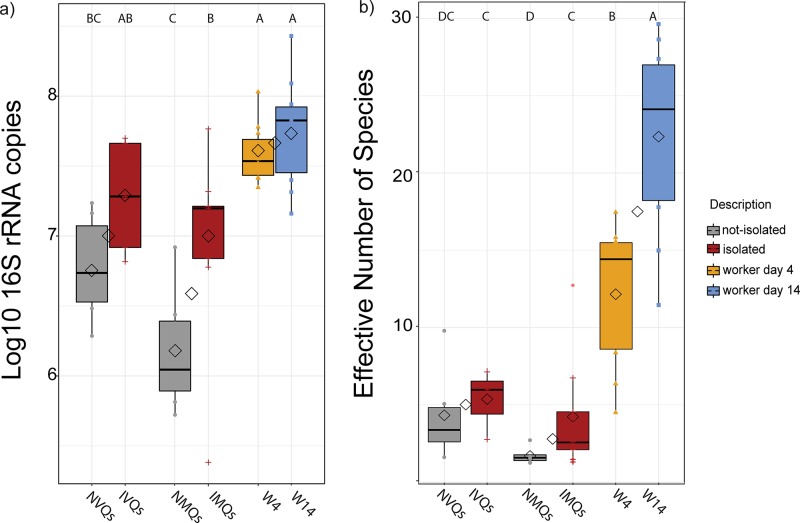
Comparison of 16SrRNA gene copy numbers and alpha diversity between sample groups. a) Total 16S rRNA gene copies for queens grouped by mating and isolation state, as well as for workers. b) Average Effective Number of Species from 10 samplings of a rarified OTU table at a depth of 1800 reads. Box plots represent quantiles, and diamonds signify the mean gene copy number within each mating state and within each isolation state. Letters above the plots denote significant differences between groups (*p*<0.05, Games-Howell multiple comparisons test).

All non-isolated queens, both virgin and mated (NVQs and NMQs), had significantly fewer average 16S rRNA gene copies and lower alpha diversity (as measured by the Effective Number of Species) compared to 4-day-old (W4s) and 14-day-old (W14s) workers (Fig 2). Also, NMQs had significantly fewer 16S rRNA gene copies and a lower Effective Number of Species than any isolated (IVQs and IMQs) queens (Fig 2A and 2B; *p*<0.05, Tukey’s HSD for 16S rRNA copies and Games-Howell multiple comparisons test for alpha diversity). IVQs had similar 16S rRNA gene copy numbers compared to either IMQs or W4s and W14s (Fig 2A; *p*>0.05, Tukey’s HSD). Thus, isolating queens during the first few days following eclosure results in a later elevation of gut bacteria numbers.

### Taxonomic analysis

**Fig 3:**
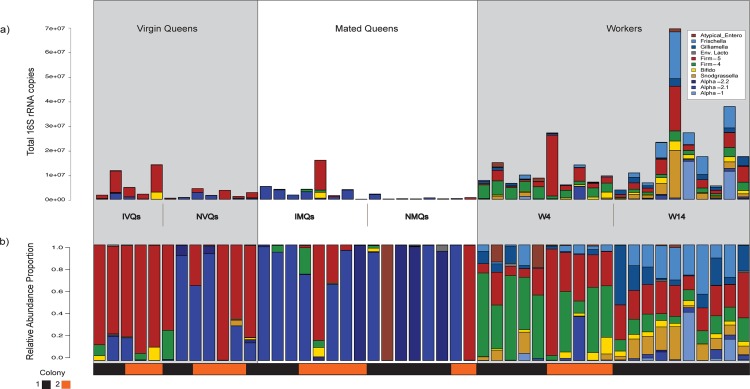
Bar plots of absolute and relative abundance of bacterial taxa per sample. a) Absolute abundance of bacterial species in queen samples subdivided by mating and isolation state and by colony of origin. The bar plots were made by multiplying the total number of 16S rRNA copies by the relative abundance proportions, and then dividing taxon-specific groups by the number of 16S rRNA operons observed in corresponding complete genomes. b) Relative abundance of bacterial species in samples subdivided by mating and isolation state and by colony of origin.

Honey bee queen gut microbial communities had compositions very different from those of workers. Queen guts were dominated by lineages of Acetobacteraceae (Alpha-2.1 and Alpha-2.2), and by Firm-5 clade lactobacilli (Fig 3). The relative proportions of typical worker core species differed between IVQs and NVQs (i.e., *Snodgrassella alvi*), as well as between IMQs and NMQs (i.e., *Gilliamella apicola*) with non-isolated queens, exhibiting higher proportions of these taxa in each mating cohort (S1 Fig, *p*<0.05, Kruskal-Wallis test). The proportions of these core species were mostly low, with all samples exhibiting <1% for these taxa except for one NVQ, which had ~5% *S*. *alvi* relative abundance. The absolute and relative abundance of these taxa are shown in S4 Table.

**Fig 4:**
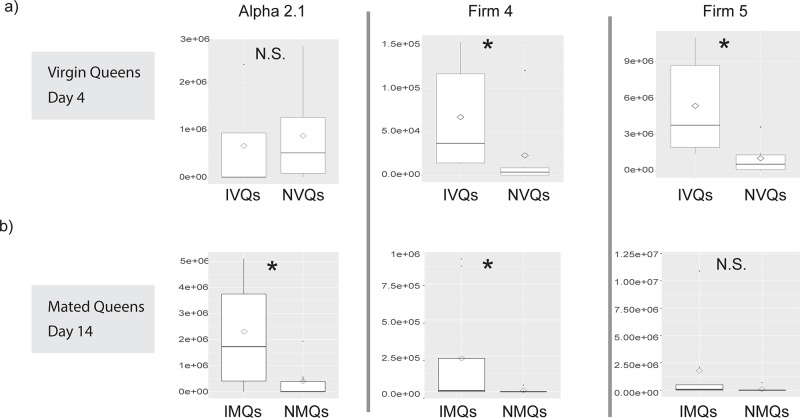
Box plots comparing the average absolute abundance of specific taxa between isolated and non-isolated groups of queens. Average absolute abundance of taxonomic lineages in queens from either a) virgin groups (collected at 4 days post eclosure) or b) mated groups (collected at 14 days post eclosure). Box plots show comparisons of taxa between isolated and non-isolated queens (* = *p*<0.05, N.S. = *p*>0.05, Kruskall-Wallis test).

Regarding absolute species abundance, Firm-4 and Firm-5 clade lactobacilli were more abundant in IVQs than in NVQs (Fig 4A; *p*<0.05, Kruskal-Wallis). Alpha-2.1, and Firm-4 lactobacilli were more abundant in IMQs compared to NMQs (Fig 4B; *p*<0.05, Kruskal-Wallis).

**Fig 5:**
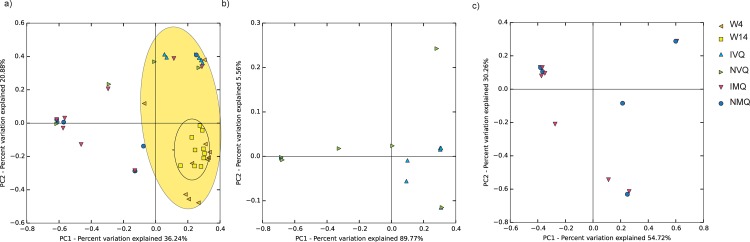
Bray-Curtis PCA plots of gut microbial community composition. PCA Plots were based on sampling OTUs at a depth of 1800 reads. a) Plot of all samples, coded by mating state. The yellow outer ellipsoid denotes the area occupied by workers and the inner ellipsoid shows dense clustering by 14-day-old workers. Significant differences were observed between workers and the mating states (mated vs. virgin) of queens (Adonis PERMANOVA, R^2^ = 0.39; F_4,43_ = 6.88; *p*< 0.001). b) Plot of virgin queens (4 days post eclosure) only. c) Plot of mated queens (14 days post eclosure) only. No significant differences were observed between isolated and non-isolated queens within the two age groups (*p*>0.05, Adonis PERMANOVA). The distances are based on an abundance-weighted Bray-Curtis analysis.

PCA of Bray-Curtis distances, which is based on the presence and abundance of OTUs, was performed for all samples. In this analysis, workers of both age classes were clustered together, and significant differences were found between workers as a group and queens (including both mating states) as a group (Fig 5). However, no significant differences were found between groups when the isolated and non-isolated states were examined within queen mating states (virgin versus mated; *p*>0.05, Adonis PERMANOVA). Thus, workers had a distinct gut microbial community whereas queens, regardless of the sampling point or isolation status, did not have distinct communities. Together, the relative and absolute abundance analyses show that isolation post eclosion had its greatest effect on the absolute size of the queen gut community rather than on its species composition, as reflected in the overall profile or presence of particular bacterial community members.

## Discussion

We found that the gut microbial communities in honey bee queens are dominated by Acetobacteraceae and lactobacilli early in a queen’s development (day 4 post emergence) and transition to mainly Acetobacteraceae lineages (mostly Alpha-2.1) as the queen ages (day 14 post emergence; Fig 3). Our study replicated the finding from previous studies, which showed that honey bee queens lack the stable core microbiota associated with workers and possess much less diverse and less consistent bacterial communities than workers of similar ages [31,32].

Isolation of virgin queens from nurses early in their life cycle (72 hours of isolation starting on day 1 post emergence) resulted in queens with larger and more diverse gut microbial communities as compared to virgin queens that were not isolated (IVQs versus NVQs). Rather than being reduced over time, this effect became more severe as the queens aged and mated (Fig 2). Indeed, NMQs sampled 14 days post eclosion had the fewest 16S rRNA gene copies of any of the sampled groups (mean = 6.16 ± 0.4 S.D. log_10_ 16S rRNA gene copies), whereas the equivalently aged isolated queens (IMQs) had significantly more 16S rRNA gene copies (mean = 6.97± 0.66 S.D. log_10_ 16S rRNA gene copies). Furthermore, absolute abundance measures showed that IVQs exhibited larger proportions of Firm-4 and Firm-5 lactobacilli, which persisted to a lesser degree in the older IMQs (Figs 3 & 4).

Our final sampling was done in young queens following the onset of reproduction. However, honey bee queens can live for several years, and sampling queens older than a few weeks could indicate whether the effect of early isolation on the queen’s microbiome persists for significant portions of the queen’s life [2].

Results from a previous study [31] resembled ours for queens that were isolated during early adulthood. In that study, bacterial communities sampled from queens increased in size as the queens matured, and mature queens had similarly sized microbiomes compared to workers from the same colony. If the queens in that study did in fact encounter a period of segregation following eclosion (which the authors of that study did not specify), then results of our study and the previous study are consistent with each other. Other potential confounding factors that may influence the size of a mature queen’s microbiome, and which could have led to differences between studies, include hygienic genetics, geography and nutrition. For instance, our study used closely related sister queens and other genotypes could potentially show different microbiome patterns. However, to date, *A*. *mellifera* workers have shown fairly consistent microbiome composition across diverse colonies and localities [42–48], suggesting that genotypic variation does not have a large effect on the honey bee gut microbiome.

We found that isolating young queens, which prevents full contact with nurses early in life, results in a subsequent elevation of the gut’s bacterial community size, the alpha diversity, and the number of guts dominated by single-taxa. Thus, full contact with nurses during the first days of adulthood seems to curtail the subsequent size of the queen’s microbiome. The underlying mechanisms for these effects are not clear, but they could involve priming of the queen’s own immune system early in adult life [65], social immunity [27], or interruption in the access to antimicrobial substances provided by nurses to young queens [3]. Social immunity potentially involves elements similar to those documented in ants and termites [66–68], including the transmission of endocrine factors for the activation of the queen’s immune system or the passage of antimicrobial peptides from nurses to queens. A recent study [69] documented the movement of dsRNA molecules between bees within a colony through the royal jelly that is transferred from nurse bees to larvae. Because this transfer continues through adulthood in adult queens, early isolation could potentially affect such transfer and alter gene expression and immune function in queens. Further examination of how queens acquire and regulate their microbiome will help us better understand how honey bee queen health can be improved in commercial queen-rearing operations.

## Supporting information

S1 Fig Average relative abundance of taxonomic lineages in queens that belonged to one of two groups: a) virgin, collected at 4 days post eclosure, or b) mated, collected at 14 days post eclosure. Boxplots show comparisons of taxa between isolated or non-isolated queens (* = *p*<0.05, Kruskall-Wallis test).
(EPS)

S2 Fig Alpha diversity rarefaction plot (Expected Number of OTUs per Sampled Reads). The plot was produced by using the *rarecurve* command in the R package *Vegan*. The trendlines are colored by treatment. The numbers correlate to samples found in S2 Table.
(EPS)

S1 Table Read processing information: a) raw read and processing statistics, b) sample read and processing statistics.
(XLSX)

S2 Table Sample specific information. (CSV)

S3 Table Summary of sample types. (XLSX)

S4 Table Taxonomic abundance tables: a) relative abundance, b) absolute abundance.
(XLSX)

S5 Table Taxonomic assignments for high abundance OTUs. (XLSX)

S1 Appendix Sequences of high abundance OTUs. (FASTA)
